# Health Warnings on Instagram Advertisements for Synthetic Nicotine E-Cigarettes and Engagement

**DOI:** 10.1001/jamanetworkopen.2024.34434

**Published:** 2024-09-13

**Authors:** Jiaxi Wu, Briana M. Trifiro, Lynsie R. Ranker, Juan Manuel Origgi, Emelia J. Benjamin, Rose Marie Robertson, Aruni Bhatnagar, Andrew C. Stokes, Ziming Xuan, Derry Wijaya, Bryan Plummer, Jennifer Cornacchione Ross, Jessica L. Fetterman, Traci Hong

**Affiliations:** 1 Annenberg School for Communication, University of Pennsylvania, Philadelphia; 2 College of Communication, Boston University, Boston, Massachusetts; 3 Department of Community Health Sciences, School of Public Health, Boston University, Boston, Massachusetts; 4 Department of Computer Science, Boston University, Boston, Massachusetts; 5 Section of Cardiovascular Medicine, Department of Medicine, Boston Medical Center, Boston University Chobanian & Avedisian School of Medicine, Boston, Massachusetts; 6 Department of Epidemiology, Boston University School of Public Health, Boston, Massachusetts; 7 American Heart Association Tobacco Regulation and Addiction Center, Dallas, Texas; 8 Department of Medicine, School of Medicine, Vanderbilt University, Nashville, Tennessee; 9 Department of Medicine, University of Louisville, Louisville, Kentucky; 10 Global Health, School of Public Health, Boston University, Boston, Massachusetts; 11 Department of Health Law, Policy & Management, School of Public Health, Boston University, Boston, Massachusetts; 12 Evans Department of Medicine and Whitaker Cardiovascular Institute, Boston University Chobanian & Avedisian School of Medicine, Boston, Massachusetts

## Abstract

**Importance:**

Synthetic nicotine is increasingly used in e-cigarette liquids along with flavors to appeal to youths. Regulatory loopholes have allowed tobacco manufacturers to use social media to target youths.

**Objective:**

To analyze the extent to which synthetic nicotine e-cigarette brands have implemented US Food and Drug Administration (FDA) health warning requirements and to evaluate the association between health warnings and user engagement on Instagram.

**Design, Setting, and Participants:**

In this cross-sectional study, posts from 25 brands were analyzed across a 14-month period (August 2021 to October 2022). A content analysis was paired with Warning Label Multi-Layer Image Identification, a computer vision algorithm designed to detect the presence of health warnings and whether the detected health warning complied with FDA guidelines by (1) appearing on the upper portion of the advertisement and (2) occupying at least 20% of the advertisement’s area. Data analysis was performed from March to June 2024.

**Exposure:**

Synthetic nicotine e-cigarette advertisement on Instagram.

**Main Outcomes and Measures:**

The outcome variables were user engagement (number of likes and comments). Negative binomial regression analyses were used to evaluate the association between the presence and characteristics of health warnings and user engagement.

**Results:**

Of a total of 2071 posts, only 263 (13%) complied with both FDA health warning requirements. Among 924 posts with health warnings, 732 (79%) displayed warnings in the upper image portion, and 270 (29%) had a warning covering at least 20% of the pixel area. Posts with warnings received fewer comments than posts without warnings (mean [SD], 1.8 [2.5] vs 5.4 [11.7] comments; adjusted incident rate ratio [aIRR], 0.70; 95% CI, 0.57-0.86; *P* < .001). For posts containing warnings, a larger percentage of the warning label’s pixel area was associated with fewer comments (aIRR, 0.96; 95% CI, 0.93-0.99; *P* = .003). Flavored posts with health warnings placed in the upper image portion received more likes than posts with warnings in the lower portion (mean [SD], 34.6 [35.2] vs 19.9 [19.2] likes; aIRR, 1.48; 95% CI, 1.07-2.06; *P* = .02).

**Conclusions and Relevance:**

In this cross-sectional study of synthetic nicotine brand Instagram accounts, 87% of sampled posts did not adhere to FDA health warning requirements in tobacco promotions. Enforcement of FDA compliant health warnings on social media may reduce youth engagement with tobacco marketing.

## Key Points

**Question:**

Do synthetic nicotine brands adhere to the Food and Drug Administration (FDA) guidelines for tobacco marketing, and how is the presence of a health warning associated with user engagement on Instagram?

**Findings:**

In this cross-sectional study of 2071 Instagram posts for 25 synthetic nicotine brands, only 263 posts (13%) adhered to FDA health warning requirements. Posts advertising flavored products received more likes and comments, and those with health warnings received fewer comments, indicating that compliance with FDA guidelines reduces user engagement.

**Meaning:**

These findings suggest that enforcing FDA-compliant health warnings on social media posts of synthetic nicotine products may reduce youth engagement with tobacco marketing on social media.

## Teaser

This cross-sectional study examines whether synthetic nicotine e-cigarette brands implemented Food and Drug Administration health warning requirements and evaluates the association between health warnings and user engagement on Instagram.

## Introduction

Synthetic nicotine products have rapidly grown in popularity since their introduction in 2020 and currently account for nearly two-thirds of all product offerings in vape shops across the US.^1^ Synthetic nicotine is a form of nicotine produced in a laboratory setting that uses chemical precursors derived from nontobacco sources.^2^ Very little is known regarding the health effects associated with synthetic nicotine use.^2^ However, synthetic nicotine is pharmacologically similar to tobacco-derived nicotine and likely has similar addictive properties.^2^ Nicotine addiction adversely affects brain development and psychosocial health in youths, and those who use e-cigarettes are at increased risk of using combustible tobacco, which has known adverse health outcomes.^3,4,5,6^

Because synthetic nicotine is not derived from tobacco, it evaded the regulatory authority of the US Food and Drug Administration (FDA) until April 2022.^7^ This regulatory loophole allowed manufacturers to flood the tobacco market with synthetic nicotine products.^8^ Youths who use tobacco are drawn to flavored e-cigarettes.^9^ Flavors like pink lemonade and cotton candy, previously banned in traditional tobacco products and cartridge-based e-cigarettes, contribute to the appeal of synthetic nicotine e-cigarettes among youths.^8,10,11^ In addition, manufacturers of synthetic nicotine e-cigarettes often market their products as *nontobacco nicotine* or *tobacco-free nicotine*, implying that synthetic nicotine products are safer than traditional tobacco products.^8,12^ This marketing strategy leads young adults to perceive synthetic nicotine products as less addictive than traditional tobacco products, fostering positive perceptions and potentially increasing trial among youths.^13^

Many manufacturers of synthetic nicotine products use social media marketing techniques on platforms such as Instagram, including the use of colorful graphics to emphasize fruit or dessert flavors.^14^ Exposure to tobacco advertising on social media is associated with a greater willingness and intention to use e-cigarettes, lower perceived danger of e-cigarette use, and increased tobacco use.^15,16,17^ In addition, engagement with content, including liking or following content shared by e-cigarette brands, is associated with e-cigarette trial among youths.^18^

Although federal laws require health warning statements to be displayed on the packaging and print advertising for tobacco products sold or distributed in the US,^19^ the current standards were developed for a pre–social media world.^20^ The gray areas surrounding federal regulation specific to social media marketing of tobacco products are particularly concerning because more than 9 in 10 teens report using the internet at least daily, with approximately one-half of US teens using Instagram daily.^21^

Our objective was to analyze the promotional content shared by synthetic nicotine brands on Instagram. We focus on Instagram because it is considered a primary source of socialization and information sharing among youths.^22,23^ Given the increasing use of synthetic nicotine e-cigarettes among youths and the regulatory loophole in social media marketing of tobacco products,^1,20^ we hypothesize that (1) the majority of promotional content shared by synthetic nicotine brands on Instagram does not adhere to FDA guidelines for health warnings,^19^ and (2) Instagram posts with warnings have lower user engagement (ie, numbers of likes and comments) than posts with no warnings.

## Methods

### Data Collection

Our sample was based on the previously identified leading 107 manufacturers of synthetic nicotine products.^8^ To be included in the sample, the synthetic nicotine brand account must (1) have been actively posting on Instagram during the data collection period, and (2) have shared at least 1 post promoting synthetic nicotine products during the study period. We identified 25 synthetic nicotine brand accounts that fit these criteria. We analyzed 2120 posts shared over a 14-month period (August 2021 to October 2022). For carousel posts with multiple images, only the first image was analyzed. The Boston University institutional review board deemed the study to not pertain to human participants; thus, our work was exempt from review and the need for informed consent, in accordance with 45 CFR §46. We followed the Strengthening the Reporting of Observational Studies in Epidemiology (STROBE) reporting guidelines.^24^

To verify the ownership of the identified synthetic nicotine brand Instagram profiles, we checked whether the profile pictures matched or were similar to the manufacturer’s branding logo on their official websites. In our sample, 76% of the synthetic nicotine brands provided a direct link from their webpage to their Instagram account. We compared each brand’s logo with their Instagram profile picture and determined whether the brand’s webpage linked to an Instagram account (eTable 1 in Supplement 1). We also collected data on the Instagram profiles’ features, such as whether there were any age restrictions to view the account, the presence of health warning statements in the account’s profile information, and whether the account self-identified as a business or a personal account.

We manually downloaded 2184 images and videos from 2120 posts, along with their accompanying comments, over a 3-week period in October 2022. We excluded 64 images that were not the first image in a carousel post. Individual posts that hid like counts were excluded from our analysis (38 posts). Engagement metrics for each post (number of likes and comments) were recorded. In line with previous research criteria that exclude posts without sufficient time for engagement,^25,26^ 6 posts were excluded from the sample because they were posted within 48 hours of data collection.

### Business and Nonbusiness Accounts

Instagram allows users to differentiate their accounts as business or nonbusiness within the account’s profile. Business accounts have access to enhanced metrics and capabilities compared with nonbusiness accounts, including insights and growth analytics.^27^ Our sample of accounts includes 13 designated as business and 12 designated as nonbusiness.

### Instagram Post Content Analysis

We coded for product flavors, use of fruit or dessert images, and vaping cues. A post featured a vaping cue if it included (1) an individual holding a product (e-cigarette or e-liquid), (2) a vape cloud, and/or (3) actual vaping behavior. Four trained coders (not coauthors of this article) completed 4 rounds of pilot coding for intercoder reliability, achieving a mean Gwet agreement coefficient of 0.94, indicating very good reliability.^28,29^ The coders then independently coded the remaining posts after establishing reliability. A list of Gwet agreement coefficient intercoder reliability coefficients appear in eTable 2 in Supplement 1. Upon establishing intercoder reliability, the coders independently coded the remaining posts.

### Warning Label Multi-Layer Image Identification

We designed the Warning Label Multi-Layer Image Identification (WaLi) computer vision algorithm^30^ to detect the presence of health warnings and whether the health warning label (1) appeared on the upper portion of the advertisement within the trim area and (2) occupied at least 20% of the advertisement’s area, per FDA guidelines for print advertisements of tobacco products.^31^ For video posts, we took still images of the first frame of all the videos in the sample to pass through WaLi. We manually validated WaLi on all collected 2184 images, 969 of which had warnings and 1215 that did not. The overall accuracy of WaLi to detect the presence of health warnings in our dataset was 99%, with recall of 98%, precision of 100%, F1 score of 99%, and Hamming Loss of 1.2%.

### Statistical Analysis

Data analysis was performed from March to June 2024. Because our outcome variables (likes and comments) are count data, we considered Poisson or negative binomial regressions. Poisson regression assumes the variance equals the mean (ie, no overdispersion), whereas negative binomial regression accommodates overdispersion.^32^ Likelihood ratio tests between the Poisson and negative binomial models indicated that negative binomial regression fit the data better (eTable 3 in Supplement 1).

Thus, we used negative binomial regression models to examine the association between the presence and characteristics of warnings and user engagement, with separate models for likes and comments. First, for all posts, we analyzed the associations of warning and flavor presence with engagement. Next, for posts promoting flavored synthetic nicotine products specifically, we examined the association of health warning presence with engagement. Then, among posts that included health warnings, we examined how warning placement (whether the warning is placed in the upper portion of the image) and warning size (percentage of pixels covered) were associated with engagement, alongside the presence of flavor. Finally, in posts advertising flavored products with health warnings, we analyzed the association between engagement and warning characteristics (placement and size). Five posts were excluded from the statistical analysis because WaLi was unable to estimate the warning area owing to black borders blending with other black pixels in the images (eFigure in Supplement 1).

All models were adjusted for follower counts, days since posting, and whether the content originated from a business account.^33,34,35^ We used random effects by brand to account for clustering of the post data within the same brand. Negative binomial models were fit using the glmmADMB package in R statistical software version 4.1.2 (R Project for Statistical Computing). The exponentiated regression coefficients in the negative binomial model are reported as adjusted incident rate ratios (aIRRs). Corresponding 95% CIs and *P* values are reported, with a 2-sided threshold of *P* < .05 for statistical significance.

## Results

### Synthetic Nicotine Brand Instagram Account Characteristics

Table 1 presents an overview of the characteristics of synthetic nicotine e-cigarette brands in the sample. Notably, 13 accounts (52%) restricted access to users younger than 21 years, and 13 accounts were classified as business accounts. In addition, 24 accounts (96%) used a brand logo that was consistent with the profile picture. Only 4 accounts (16%) featured health warnings in their profiles. Age restrictions were noted in the profiles of 14 accounts (56%), and 18 accounts (72%) included links to the manufacturer’s website.

**Table 1.** Characteristics of the 25 Synthetic Nicotine Brand Instagram Accounts

Characteristic	Brands, No. (%)
Account characteristics	
Unable to be viewed by users aged <21 y	13 (52)
Classified as a business account	13 (52)
Profile picture matched brand’s logo	24 (96)
Include health warnings in posts	19 (76)
Account profile characteristics	
Presence of a health warning^a^	4 (16)
Presence of age restrictions	14 (56)
Presence of a link to the website	18 (72)

^a^ An Instagram account profile is the area under an account’s username that shares details about the user or brand. This space often includes brand descriptions, contact information, and links to brand webpages.

### Descriptive Results for Flavor, Presence of People, and Vaping Cues in Posts

Characteristics of the content shared by synthetic nicotine brand Instagram accounts are shown in Table 2. Overall, 1523 posts (74%) promoted a flavored product, of which 1326 (64%) promoted a product marketed as clove, spice, candy, fruit, chocolate, alcohol, or other sweet flavors. Nearly 11% of posts (222 posts) promoted a concept flavor, such as pink or cotton fluff, whereas 6% of posts (118 posts) promoted a mint or menthol flavored product. The majority of posts (1486 posts [72%]) did not feature people and 431 posts (21%) featured 1 or more vaping cues.

**Table 2.** Characteristics of Posts Shared by Synthetic Nicotine Brand Instagram Accounts

Characteristic	Posts, No. (%) (N = 2071)
Content type	
Image	1759 (85)
Video	312 (15)
Characteristics of content	
Presence of an e-cigarette	977 (47)
Presence of an e-liquid pod or cartridge	924 (45)
Posts without people	1486 (72)
Presence of ≥2 people	288 (14)
Vaping cues	
Posts contain at least 1 vaping cue	431 (21)
Person holding or handling an e-liquid, liquid pod, e-cigarette, vaping device, or vape pen in their hand (without puffing and inhaling)	387 (19)
Actual vaping behavior (puffing and inhaling of an e-cigarette, vaping device, or vape pen)	134 (6)
Image of a cloud or puff of aerosol	181 (9)
Flavored products	
Posts promote at least a flavor	1523 (74)
Image of fruit	385 (19)
Image of dessert	176 (9)
Post promotes a mint or menthol flavored product	118 (6)
Post promotes a clove, spice, candy, fruit, chocolate, alcohol, or other sweet-flavored product	1326 (64)
Post promotes a concept flavored product	222 (11)
Post setting	
Setting of post is outdoors	482 (23)
Setting of post is at a party or social gathering	36 (2)

### Health Warnings

The proportion of posts featuring health warnings by brand is available in eTable 4 in Supplement 1. Among posts in our sample, 45% (924 posts) were determined via WaLi to have a health warning. Among posts where a warning was detected, the health warning was in the upper portion of the image for 79% of posts (732 posts), and 29% of posts (270 posts) had a health warning that occupied at least 20% of the pixel area. Only 13% of posts from our entire sample (263 posts) met both FDA guidelines for health warnings.

### Instagram User Engagement

Across all 2071 posts from 25 brands, the mean (SD) like count was 46.8 (82.0), and the mean (SD) comment count was 3.8 (9.1). eTable 5 in Supplement 1 contains the descriptive results of post engagement across the analyzed samples. In negative binomial models adjusted for follower counts, business status, and days on Instagram, posts with health warnings received fewer comments than posts without health warnings (mean [SD], 1.8 [2.5] vs 5.4 [11.7] comments; aIRR, 0.70; 95% CI, 0.57-0.86; *P* < .001). Posts advertising flavored products received more likes (aIRR, 1.12; 95% CI, 1.04-1.21; *P* = .002) and comments (aIRR, 1.16; 95% CI, 1.04-1.30; *P* = .008) than posts without a flavored product. The presence of a health warning was not associated with the number of likes a post received (aIRR, 0.95; 95% CI, 0.83-1.08; *P* = .42) (Table 3).

**Table 3.** Effect Estimates of Negative Binomial Regression Model: Association of Health Warning Properties With Number of Likes and Comments for All Posts^a^

Variable	Likes				Comments			
	Coefficient	SE	*P* value	aIRR (95% CI)	Coefficient	SE	*P* value	aIRR (95% CI)
All posts (n = 2071)								
Intercept	2.60	0.27	<.001	13.46 (7.87-23.02)	−0.04	0.29	.88	0.96 (0.54-1.70)
Follower count per thousand	0.02	0.004	<.001	1.02 (1.01-1.03)	0.01	0.004	<.001	1.01 (1.005-1.022)
Days on Instagram	−0.0002	0.0001	.17	0.9998 (0.9996-1.0001)	0.0008	0.0002	<.001	1.0008 (1.0003-1.0012)
From a business account	0.10	0.31	.75	1.10 (0.60-2.05)	0.44	0.33	.18	1.55 (0.81-2.94)
Presence of health warning	−0.05	0.07	.42	0.95 (0.83-1.08)	−0.36	0.10	<.001	0.70 (0.57-0.86)
Presence of a flavor	0.12	0.04	.002	1.12 (1.04-1.21)	0.15	0.06	.008	1.16 (1.04-1.30)
Posts promoting flavored synthetic nicotine products (n = 1523)								
Intercept	2.70	0.31	<.001	14.81 (8.03-27.28)	−0.08	0.30	.80	0.93 (0.51-1.68)
Follower count per thousand	0.02	0.01	<.001	1.02 (1.01-1.03)	0.02	0.01	<.001	1.02 (1.01-1.03)
Days on Instagram	−0.0002	0.0002	.27	0.9998 (0.9995-1.0001)	0.001	0.0002	<.001	1.0009 (1.0004-1.0014)
From a business account	0.08	0.35	.82	1.08 (0.55-2.13)	0.45	0.33	.17	1.57 (0.83-3.00)
Presence of health warning	−0.04	0.08	.64	0.96 (0.83-1.12)	−0.35	0.11	.002	0.71 (0.57-0.88)

Abbreviation: aIRR, adjusted incidence rate ratio.

^a^ Estimates come from a single model rather than separate models for each independent variable. All models were adjusted for brand follower counts, days since post, and random effects of 25 brands to account for the clustering of the posts within the same brand.

Among 924 posts with health warnings, a greater proportion of the warning label’s pixel area was associated with fewer comments (aIRR, 0.96; 95% CI, 0.93-0.99; *P* = .003), with no significant association found for likes (aIRR, 0.99; 95% CI, 0.98-1.003; *P* = .13). A health warning in the upper portion of the post’s image was not associated with numbers of likes (aIRR, 1.28; 95% CI, 0.96-1.70; *P* = .09) or comments (aIRR, 1.50; 95% CI, 0.87-2.58; *P* = .15) (Table 4).

**Table 4.** Effect Estimates of Negative Binomial Regression Model: Association of Health Warning Properties With Number of Likes and Comments for Posts With Health Warnings^a^

Variable	Likes				Comments			
	Coefficient	SE	*P* value	aIRR (95% CI)	Coefficient	SE	*P* value	aIRR (95% CI)
All posts with health warnings (n = 924)								
Intercept	2.34	0.28	<.001	10.43 (6.01-18.09)	0.03	0.42	.95	1.03 (0.45-2.36)
Follower count	0.01	0.004	<.001	1.01 (1.01-1.02)	0.01	0.01	.02	1.01 (1.002-1.024)
Days on Instagram	0.005	0.0002	.002	1.0005 (1.0002-1.0008)	0.001	0.0003	<001	1.0011 (1.0005-1.002)
From a business account	0.38	0.27	.15	1.47 (0.87-2.49)	0.37	0.36	.31	1.44 (0.71-2.93)
Presence of a flavor	0.14	0.07	.04	1.15 (1.01-1.32)	0.07	0.15	.61	1.08 (0.81-1.43)
Health warning in upper portion of image	0.25	0.15	.09	1.28 (0.96-1.70)	0.40	0.28	.15	1.50 (0.87-2.58)
Percentage of health warning pixel area	−0.01	0.01	.13	0.99 (0.98-1.003)	−0.04	0.14	.003	0.96 (0.93-0.99)
Posts promoting flavored products with health warnings (n = 851)								
Intercept	2.46	0.28	<.001	11.70 (6.81-20.11)	0.07	0.41	.87	1.07 (0.48-2.39)
Follower count	0.01	0.004	<.001	1.015 (1.006-1.023)	0.01	0.01	.02	1.01 (1.002-1.02)
Days on Instagram	0.0005	0.0002	.006	1.0005 (1.0001-1.0008)	0.001	0.0004	<.001	1.001 (1.0005-1.002)
From a business account	0.37	0.27	.17	1.45 (0.85-2.45)	0.36	0.36	.33	1.43 (0.70-2.90)
Health warning in upper portion of image	0.39	0.17	.02	1.48 (1.07-2.06)	0.53	0.31	.08	1.70 (0.93-3.08)
Percentage of health warning pixel area	−0.01	0.01	.07	0.99 (0.97-1.001)	−0.04	0.01	.003	0.96 (0.93-0.99)

Abbreviation: aIRR, adjusted incidence rate ratio.

^a^ Estimates come from a single model rather than separate models for each independent variable. All models were adjusted for brand follower counts, days since post, and random effects of 25 brands to account for the clustering of the posts within the same brand.

### Health Warnings and User Engagement for Posts Promoting Flavors

Among 1523 posts that advertised flavored synthetic nicotine products, the presence of a health warning was associated with fewer comments (aIRR, 0.71; 95% CI, 0.57-0.88; *P* = .002) but not associated with likes (aIRR, 0.96; 95% CI, 0.83-1.12; *P* = .64) (Table 3). When focusing on 851 posts promoting flavored products with health warnings, a higher percentage of the warning label’s pixel area was associated with fewer comments (aIRR, 0.96; 95% CI, 0.93-0.99; *P* = .003). A health warning in the upper portion of the post’s image was associated with increased numbers of likes (mean [SD], 34.6 [35.2] vs 19.9 [19.2] likes; aIRR, 1.48; 95% CI, 1.07-2.06; *P* = .02) (Table 4).

## Discussion

This cross-sectional study found that most synthetic nicotine brand Instagram posts (87%) do not adhere to FDA health warning requirements for tobacco advertising. Our findings show an association between the presence of a health warning and user engagement on Instagram. Posts that feature health warnings received fewer comments. Warnings covering a larger percentage of the pixel area received fewer comments.

Although there are not yet social media–specific guidelines for tobacco marketing, our findings suggest that the implementation of FDA guidelines for warning labels in promotional content for synthetic nicotine products negatively affects social media engagement. Larger warning sizes in particular are associated with reduced post engagement.

Although our study did not find an association between the presence of a health warning and number of likes, our work shows a clear association between health warnings and comments. Although it is easy to like a post, a user needs to exert more effort to comment on a post, suggesting more engagement.^36^

Consistent with prior research demonstrating the appeal of flavors in e-cigarettes to youths,^14,37,38,39,40,41^ our study found that synthetic nicotine brands on Instagram frequently promote fruit and dessert flavors, which were associated with higher likes and comments. A majority (74%) of posts featured flavored products, often showcased with visually appealing images of fruits or desserts. Our findings reveal that FDA-compliant health warnings were negatively associated with comments on posts promoting flavored synthetic nicotine products, suggesting that health warnings may effectively reduce engagement with such promotional content of synthetic nicotine e-cigarettes.

Unexpectedly, placing a health warning in the upper portion of posts promoting flavored products was associated with increased likes. One possible explanation is that when warnings are not displayed in the upper portion of the post, they are likely to be placed on the product in the post. Warnings on products may lead people to directly associate nicotine’s health risks with the product, possibly resulting in fewer likes for the post. Therefore, more research is necessary to explore how placing warnings in social media marketing of synthetic nicotine products (eg, on the products) influences user engagement and perceptions of synthetic nicotine e-cigarettes.

Research^42,43,44,45^ shows that vaping in advertisements for e-cigarettes can serve as a cue to individuals who use tobacco products, increasing the urge to vape and smoke combustible cigarettes. Exposure to vaping cues has been associated with lower levels of intention to quit or abstain from tobacco use.^42,43,44,45^ Although the majority of posts did not feature people, of those that did, models were often engaged in vaping cues. This finding shows how synthetic nicotine brands model vaping behavior for social media audiences.

Current federal regulations mandate health warnings on print and image-based advertisements for tobacco products, but it remains unclear whether these regulations apply to all forms of social media marketing. Similar to prior research,^31^ our study indicates that including health warnings is associated with reduced social media engagement with social media tobacco promotions. The use of health warnings can convey the risks associated with tobacco products to social media users, which may ultimately deter uptake and use of tobacco products.^46,47,48^ Given the link between exposure to e-cigarette marketing on social media and youth tobacco initiation,^16,49,50,51,52^ our findings suggest that clarifying regulations for implementing health warnings in promotional content for synthetic nicotine products on social media may deter engagement with such brand content.

### Limitations

This study has limitations that should be acknowledged. We relied on data acquired from a social media platform, which may introduce inherent biases. To mitigate this challenge, we conducted a thorough search for synthetic nicotine brands to ensure diverse and representative samples. We also used objective machine learning methods to measure the presence and properties of health warnings. Our findings may not be generalizable to other tobacco products, brands, or social media platforms. In addition, our study only analyzed engagement metrics including likes and comments. Future research should further explore the sentiment of comments to better understand how health warnings affect audience perceptions of brands and products. We do not know the tobacco use status of the Instagram users who engaged with the posts in our sample, nor do we know the ages of users exposed to these posts; therefore, we cannot determine or argue any causal effects of the presence of health warnings on user outcomes, such as tobacco use or intention to quit.

Our study did not analyze the exact language used in health warnings. The language used in the sampled warnings varied among brands. Future research would benefit from analyzing specific language used in health warnings to understand how brands are conveying potential harms associated with using tobacco products. Because WaLi relies on a modular algorithm, future research could benefit from adapting the Optical Character Recognition model language in order to identify warnings in different languages. In addition, the time period of our data collection falls before and after the regulatory shift in April 2022 that brought synthetic nicotine under the FDA’s regulatory purview. Although our study does not analyze how posting behavior of synthetic nicotine brands changed during this time period because it is outside the scope of our project, future work would benefit from a comprehensive understanding of whether this regulatory shift led to changes in the social media strategies of synthetic nicotine brands.

## Conclusions

In the current study, most promotional Instagram content posted by synthetic nicotine brands did not adhere to federal regulations requiring health warnings on advertisements. Posts that included health warnings and a larger warning size received less user engagement. Health warnings may lessen user engagement, which could deter youth uptake and use of synthetic nicotine products.

## Notes

Supplement 1. **eTable 1.** Instagram account validation procedure **eTable 2.** Intercoder reliability—Gwet’s AC1 coefficients **eTable 3.** Number of parameters, log-likelihood, and Akaike Information Criterion for the negative binomial and Poisson regression models with Instagram content from synthetic nicotine brands **eTable 4.** Proportion of posts featuring warning labels by brand **eTable 5.** Descriptive results of post engagement **eFigure.** Data inclusion criteria for statistical analysis

Supplement 2.
Data Sharing Statement
