# Supplementary material for: Health Warnings on Instagram Advertisements for Synthetic Nicotine E-Cigarettes and Engagement
Source: JAMA Netw Open. 2024 Sep 13;7(9):e2434434. doi: 10.1001/jamanetworkopen.2024.34434 (PMC11400217; doi:10.1001/jamanetworkopen.2024.34434)
Supplement: Supplement 1. — eTable 1. Instagram account validation procedure eTable 2. Intercoder reliability—Gwet’s AC1 coefficients eTable 3. Number of parameters, log-likelihood, and Akaike Information Criterion for the negative binomial and Poisson regression models with Instagram content from synthetic nicotine brands eTable 4. Proportion of posts featuring warning labels by brand eTable 5. Descriptive results of post engagement eFigure. Data inclusion criteria for statistical analysis [file jamanetwopen-e2434434-s001.pdf]

Supplemental Online Content

Wu J, Trifiro BM, Ranker LR, e al. Health warnings on Instagram advertisements of synthetic nicotine e-cigarettes and engagement. *JAMA Netw Open*. 2024;7(9):e2434434.
doi:10.1001/jamanetworkopen.2024.34434

eTable 1. Instagram account validation procedure
eTable 2. Intercoder reliability—Gwet's AC1 coefficients
eTable 3. Number of parameters, log-likelihood, and Akaike Information Criterion for the negative binomial and Poisson regression models with Instagram content from synthetic nicotine brands
eTable 4. Proportion of posts featuring warning labels by brand
eTable 5. Descriptive results of post engagement
eFigure. Data inclusion criteria for statistical analysis

This supplemental material has been provided by the authors to give readers additional information about their work.

© 2024 Wu J et al. *JAMA Network Open.*

eTable 1: Instagram account validation procedure
All links checked on March 9, 2023

Company name	Instagram handle	Link to manufacturer website in Instagram account bio	Brand homepage connects to Instagram account
Bantam	@bantam_vape	Yes	Yes
Cloud Nurdz	@cloudnurdz	Yes	Yes
Dinner Lady	@vapedinnerlady	Yes	Yes
Dollar E-Juice Club	@thedollarejuiceclub	Yes via Linktree	No
Exotic Bar	@exoticbarofficial	No	No
Five Pawns	@fivepawns	Yes	Yes
Frisco Vapor	@friscovapor	No	No
Fryd	@frydeliquids	Yes	Yes
Fully Loaded	@loaded.worldwide	Yes	Yes
Geek Bar	@geekbarvape	Yes	Yes
Glamee Nova	@glamee_vape	Yes	No
Ignite	@ignitecbd	Yes	Yes
Juice Roll Upz	@juicerollupz	Yes	Yes
KangVape	@kangvapecig	No	Yes
Kilo Revival	@kiloeliquids	Yes	Yes
Lost Art Liquids	@lostartliquids	Yes	Yes
Monster Bar	@monstervapelabs	Yes	Yes
Pod Juice	@podjuicesalts	No	No
Pop Clouds	@popclouds	Yes	Yes
Puff Labs	@puffxtrax	Yes	Yes
Rx Vape	@rxvapeco	Yes	Yes
Sattwa	@sattwatech	Yes	Yes
Silverback	@silverbackjuiceco	Yes via Linktree	Yes
Steam Engine Nunu	@steamengineusa	Yes via Linktree	Yes
SWFT	@swftbar	Yes	No

© 2024 Wu J et al. *JAMA Network Open.*

eTable 2: Intercoder reliability—Gwet's AC1 coefficients

Variable	Gwet's AC1
Instagram Content Type	0.98
Carousel	0.91
Presence of an electronic cigarette, e-cigarette, vaping device or vape pen	0.8
Presence of an e-liquid pod or e-liquid cartridge	0.93
Presence of a person holding or handling an e-liquid, liquid pod, electronic cigarette, e-cigarette, vaping device or vape pen in their hand	0.95
Presence of actual vaping behavior (puffing and inhaling of an electronic cigarette, e-cigarette, vaping device, or vape pen)	0.98
Presence of an image of a smoke cloud or puff of vapor	0.99
Presence of an image of a fruit	0.97
Presence of an image of a dessert	0.96
Presence of a product flavored to taste like menthol or mint	0.99
Presence of a product flavored to taste like clove, spice, candy, fruit, chocolate, alcohol or other sweets	0.88
Number of people shown in post	0.92
Presence of a synthetic nicotine product	0.96
Presence of a tobacco nicotine product	0.88
Presence of an outdoor setting	0.84
Presence of a party setting	0.99
Presence of a concept flavor	0.98

© 2024 Wu J et al. *JAMA Network Open.*

Selection between Negative Binomial Regression and Poisson Regression Models

Likelihood ratio tests between negative binomial and Poisson regressions showed significant results across all models, indicating that negative binomial regression, with higher log-likelihood values, fit the data better. Additionally, negative binomial regression models consistently reported lower Akaike Information Criterion (AIC) values, further confirming the suitability of negative binomial models over Poisson regression models (eTable 3).

eTable 3. Number of parameters, log-likelihood, and Akaike Information Criterion for the negative binomial and Poisson regression models with Instagram content from synthetic nicotine brands.

Model	Sample	Outcome variable	Predictors	Goodness of Fit Test	Likelihood-Ratio Test	
				Akaike Information Criterion (AIC)	Chisq Statistic	*P* value
Negative binomial	All posts (N = 2,071)	Likes	Presence of health warning + presence of a flavor	17722	17336	**< .001**
Poisson				35055		
Negative binomial	All posts (N = 2,071)	Comments	Presence of health warning + presence of a flavor	8435	1561	**< .001**
Poisson				9994		
Negative binomial	Posts promoting flavored synthetic nicotine products (N = 1,523)	Likes	Presence of health warning	12999	8840	**< .001**
Poisson				21837		
Negative binomial	Posts promoting flavored synthetic nicotine products (N = 1,523)	Comments	Presence of health warning	6141	1003	**< .001**
Poisson				7142		
Negative binomial	All posts with health warnings (n=924)	Likes	Presence of a flavor + warning placement + warning size	7314	5812	**< .001**
Poisson				13124		
Negative binomial	All posts with health warnings (n=924)	Comments	Presence of a flavor + warning placement + warning size	3136	335	**< .001**
Poisson				3469		
Negative binomial	Posts promoting flavored products with health warnings (n=851)	Likes	Warning placement + warning size	6763	4991	**< .001**
Poisson				11752		
Negative binomial	Posts promoting flavored products with health warnings (n=851)	Comments	Warning placement + warning size	2902	308	**< .001**
Poisson				3208		

Note. All models were adjusted for brand follower counts, days since post, business status, and random effects of 25 brands to account for the clustering of the posts within the same brand.

© 2024 Wu J et al. *JAMA Network Open.*

eTable 4: Proportion of posts featuring warning labels by brand

Brand	Instagram handle	Posts with warning labels within all brand posts, n/N(%)
Bantam	@bantam_vape	34/37 (92%)
CloudNurdz	@cloudnurdz	187/189 (99%)
Dollar E-Juice Club	@thedollarejuiceclub	142/148 (96%)
Dinner Lady	@vapedinnerlady	0/121
Exotic Bar	@exoticbarofficial	0/16
Five Pawns	@fivepawns	0/88
Frisco Vapor	@friscovapor	0/1
Fryd	@frydeliquids	20/20 (100%)
Geek Bar	@geekbarvape	12/127 (9%)
Glamee Nova	@glamee_vape	6/59 (10%)
Ignite	@ignitecbd	0/3
Juice Rollupz	@juicerollupz	185/188 (98%)
Kang Vape	@kangvapecig	1/37 (3%)
Kilo Revival	@kiloeliquids	10/21 (48%)
Fully Loaded	@loaded.worldwide	6/279 (2%)
Lost Art Liquids	@lostartliquids	49/49 (100%)
Monster Bar	@monstervapelabs	129/139 (93%)
Pod Juice	@podjuicesalts	12/18 (67%)
Pop Clouds	@popclouds	61/63 (97%)
Puff Labs	@puffxtrax	7/113 (6%)
Rx Vape	@rxvapeco	0/1
Sattwa	@sattwatech	1/33 (3%)
Silverback	@silverbackjuiceco	15/15 (100%)
Steam Engine	@steamengineusa	23/279 (8%)
SWFT	@swftbar	24/27 (89%)
**Total**		**924/2071 (45%)**

© 2024 Wu J et al. *JAMA Network Open.*

eTable 5. Descriptive results of post engagement

		Likes			Comments		
		**M (SD)**	**Median**	**IQR**	**M (SD)**	**Median**	**IQR**
**All posts (N = 2,071)**	Posts with warnings (n = 924)	31.3 (32.3)	20	21	1.8 (2.5)	1	2
	Posts without warnings (n = 1,147)	59.2 (104.6)	29	56	5.4 (11.7)	2	5
**Flavored posts (n = 1,523)**	Posts with warnings (n = 851)	31.7 (33.2)	19	21	1.8 (2.5)	1	2
	Posts without warnings (n = 672)	64.1 (118.1)	34	64	5.9 (11.8)	3	6
**Posts with warnings (n = 924)**	Health warning in upper portion of image (n = 732)	34.3 (34.4)	21	25	2.0 (2.7)	1	3
	Health warning in lower portion of image (n = 192)	19.5 (18.5)	15	13	1.2 (1.8)	1	2
**Flavored posts with warnings (n = 851)**	Health warning in upper portion of image (n = 682)	34.6 (35.2)	21	26	2.0 (2.6)	1	3
	Health warning in lower portion of image (n = 169)	19.9 (19.2)	15	13	1.2 (1.9)	1	2

© 2024 Wu J et al. *JAMA Network Open.*

eFigure: Data inclusion criteria for statistical analysis

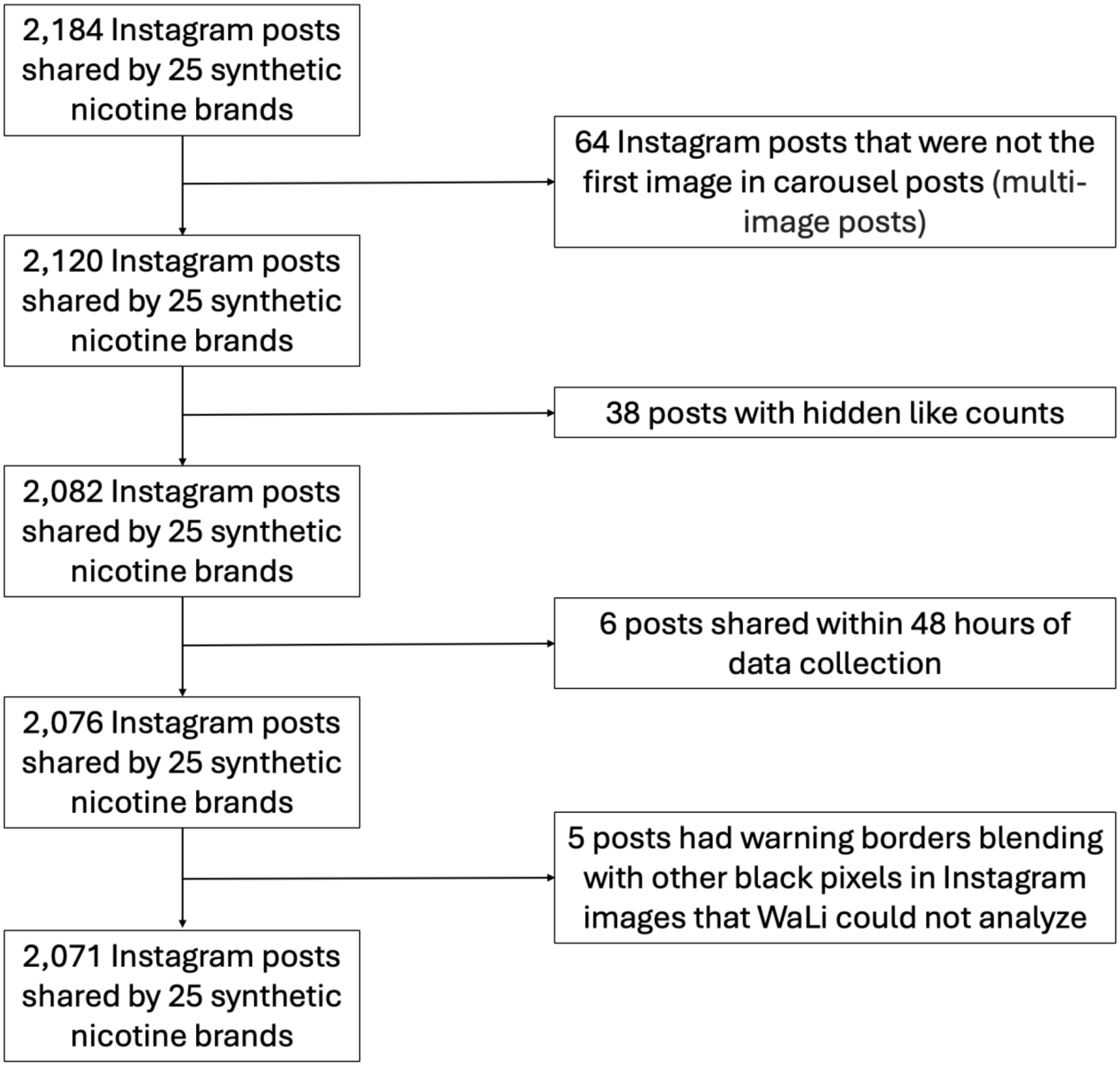

© 2024 Wu J et al. *JAMA Network Open.*

© 2024 Wu J et al. *JAMA Network Open.*
