# Supplementary material for: Health Warnings on Instagram Advertisements for Synthetic Nicotine E-Cigarettes and Engagement
Source: JAMA Netw Open. 2024 Sep 13;7(9):e2434434. doi: 10.1001/jamanetworkopen.2024.34434 (PMC11400217; doi:10.1001/jamanetworkopen.2024.34434)
Supplement: Supplement 2. — Data Sharing Statement [file jamanetwopen-e2434434-s002.pdf]

Data Sharing Statement

Wu. Health Warnings on Instagram Advertisements for Synthetic Nicotine E-Cigarettes and Engagement. *JAMA Netw Open*. Published September 13, 2024.
doi:10.1001/jamanetworkopen.2024.34434

Data
Data available: Yes
Data types: Data (not involving human participants)
How to access data: Instagram posts available upon request.
When available: With publication

Supporting Documents
Document types: Statistical/analytic code
How to access documents: https://github.com/JMOriggi/Warning-Label-Multi-Layer-Image-Identification
When available: With publication

Additional Information
Who can access the data: Data may be requested from the corresponding author, accompanied by a plan for its intended use. Data sharing will be subject to compliance with local, state, and federal laws and regulations, and may require approval from the appropriate institutional review board for human subjects.
Types of analyses: purpose of research
Mechanisms of data availability: in a repository
